# Comparison of commonly used solid tumor targeted gene sequencing panels for estimating tumor mutation burden shows analytical and prognostic concordance within the cancer genome atlas cohort

**DOI:** 10.1136/jitc-2020-000613

**Published:** 2020-03-26

**Authors:** Nicholas Bevins, Shulei Sun, Zied Gaieb, John A Thorson, Sarah S Murray

**Affiliations:** 1 Pathology, UC San Diego, La Jolla, California, USA; 2 Chemistry and Biochemistry, UC San Diego, La Jolla, California, USA

**Keywords:** tumor biomarkers, translational medical research, immunotherapy, genetic markers

## Abstract

**Background:**

Tumor mutation burden (TMB) is a biomarker frequently reported by clinical laboratories, which is derived by quantifying of the number of single nucleotide or indel variants (mutations) identified by next-generation sequencing of tumors. TMB values can inform prognosis or predict the response of a patient’s tumor to immune checkpoint inhibitor therapy. Methods for the calculation of TMB are not standardized between laboratories, with significant variables being the gene content of the panels sequenced and the inclusion or exclusion of synonymous variants in the calculations. The impact of these methodological differences has not been investigated and the concordance of reported TMB values between laboratories is unknown.

**Methods:**

Sequence variant lists from more than 9000 tumors of various types were downloaded from The Cancer Genome Atlas. Variant lists were filtered to include only appropriate variant types (ie, non-synonymous only or synonymous and non-synonymous variants) within the genes found in five commonly used targeted solid tumor gene panels as well as an in-house gene panel. Calculated TMB was paired with corresponding overall survival (OS) data of each patient.

**Results:**

Regression analysis indicates high concordance of TMB as derived from the examined panels. TMB derived from panels was consistently and significantly lower than that derived from a whole exome. TMB, as derived from whole exome or the examined panels, showed a significant correlation with OS in the examined data.

**Conclusions:**

TMB derived from the examined gene panels was analytically equivalent between panels, but not between panels and whole-exome sequencing. Correlation between TMB and OS is significant if TMB method-specific cut-offs are used. These results suggest that TMB values, as derived from the gene panels examined, are analytically and prognostically equivalent.

## Introduction

It has been recognized for more than a century that the immune system possesses an ability to recognize cancer cells as foreign, despite their origins as transformed native cells, and to subsequently destroy them.^1^ Advances in molecular biology have created novel methods to augment the immune system’s ability to recognize cancer, leading to many treatments currently available for clinical use.^2^ Collectively, these treatment methods are referred to as immunotherapy.

James Allison and Tasuku Honju recently shared the Nobel Prize for characterizing the ‘immune checkpoint’ molecular interactions of CTLA4 and PD-1/PD-L1, leading to the development of a specific type of immunotherapy.^3^ Monoclonal antibodies targeting immune checkpoint signaling pathways have become a widely used therapeutic strategy. As of 2019, there are multiple Food and Drug Administration (FDA)approved therapeutics targeting checkpoint inhibitor associated mechanisms and others in clinical trials.^4^ Despite their relative novelty, checkpoint inhibitors have quickly gained clinical popularity because they are efficacious in multiple cancer types with a favorable safety profile.

Immune checkpoint inhibitors block a tumor’s molecular ability to mask itself from the immune system, thereby exposing tumor cells to the cytotoxic effects of immune effector cells.^5^ As tumor cells ‘evolve’ from normal cells they consequently take on characteristics that allow the immune system to recognize them as foreign. Under selection from constant immune surveillance, individual tumor clones express checkpoint molecules that act as a strong ‘normal’ signal and thus mask the tumor from immune surveillance. Checkpoint inhibitors disrupt these masking signals.

Not all tumors evade the immune system through identical molecular mechanisms. Heterogeneous mechanisms of immune evasion result in clinical observations that checkpoint inhibitors are not efficacious in all tumor types or in all patients with a particular tumor type. Thus, several biomarkers have been developed in an effort to identify those patients likely to have a clinically meaningful response to checkpoint inhibitor therapy.^6^

Tumor mutation burden (TMB) is a biomarker with significant recent interest.^6^ It is derived from analysis of next-generation sequencing (NGS) of tumors and defined as the total number of somatic coding variants observed in a tumor divided by the amount of coding sequence acquired in mega-bases. Many recent retrospective studies have shown that higher TMB values are correlated with improved response rates and survival times with immune checkpoint inhibitor treatment (table 1). Of note, reported TMB calculation methods differ in panel composition and inclusion of all coding variants (including synonymous variants).

**Table 1:** Publications correlating increased TMB with response to PD-1/PD-L1 therapy

Indication	Therapy	TMB cut-off	Panel	Variant types included	Ref.
Melanoma	Ipilimumab	>2.5/ Mb*	WES	Non-synonymous	31
NSCLC	Pembrolizumab	>4.5/ Mb*	WES	Non-synonymous	32
NSCLC	Nivolumab+Ipilimumab	>4.0/ Mb*	WES	Non-synonymous	33
NSCLC	Nivolumab+Ipilimumab	>10/ Mb	FM	All coding	34–36
NSCLC	Anti-PD-1 or -PD-L1	Descriptive	MSK	Non-synonymous	37
Melanoma	Ipilimumab	Descriptive	WES	Non-synonymous	38
Urothelial	Atezolizumab	Descriptive	FM	All coding	39
NSCLC	Nivolumab	>6.2/ Mb*	WES	Non-synonymous	40
Multiple	Anti- CTLA4/PD-1/PD-L1	>20/ Mb	FM	All coding	13
Multiple	Anti- CTLA4/PD-1/PD-L1	Tumor dependent	MSK	Non-synonymous	41

*39.4 Mb/exome.

FM, foundation medicine foundation one CDx assay; MSK, memorial sloan kettering IMPACT assay; NSCLC, non-small-cell lung cancer; TMB, tumor mutation burden; WES, whole-exome sequencing.

Despite the increased clinical utilization of TMB as a biomarker, methods for calculating TMB are not currently harmonized between laboratories (although efforts at harmonization are underway).^7^ Molecular characterization of tumors is a complex and resource intensive endeavor, making method harmonization a daunting undertaking. However, the impact of changing the fundamental parameters of variant inclusion (the numerator of TMB) and sequencing area (the denominator of TMB) can be tested by in silico analyses of large, publicly available data sets such as The Cancer Genome Atlas (TCGA). Here, we report in silico simulations of six molecular profiling products, including our institution’s in-house NGS solid tumor molecular profiling method. Additionally, we correlate simulation based estimates of TMB with survival data obtained in the absence of checkpoint inhibitor therapy to observe the correlation between survival and TMB.

## Methods

### In Silico determination of TMB from TCGA

Variant Caller Format files (VCFs) created by the Somatic Sniper variant calling algorithm were downloaded from TCGA through the National Cancer Institute’s Genomic Data Commons portal (portal.gdc.cancer.gov). Per TCGA published methods, these VCFs were produced by sequencing tumor derived and paired blood derived whole exome libraries at an average of 100x depth.^8^ Minimum variant allele fraction is based on read depth at the variant site as described in the Somatic Sniper algorithm.^9^

**Figure 1:**
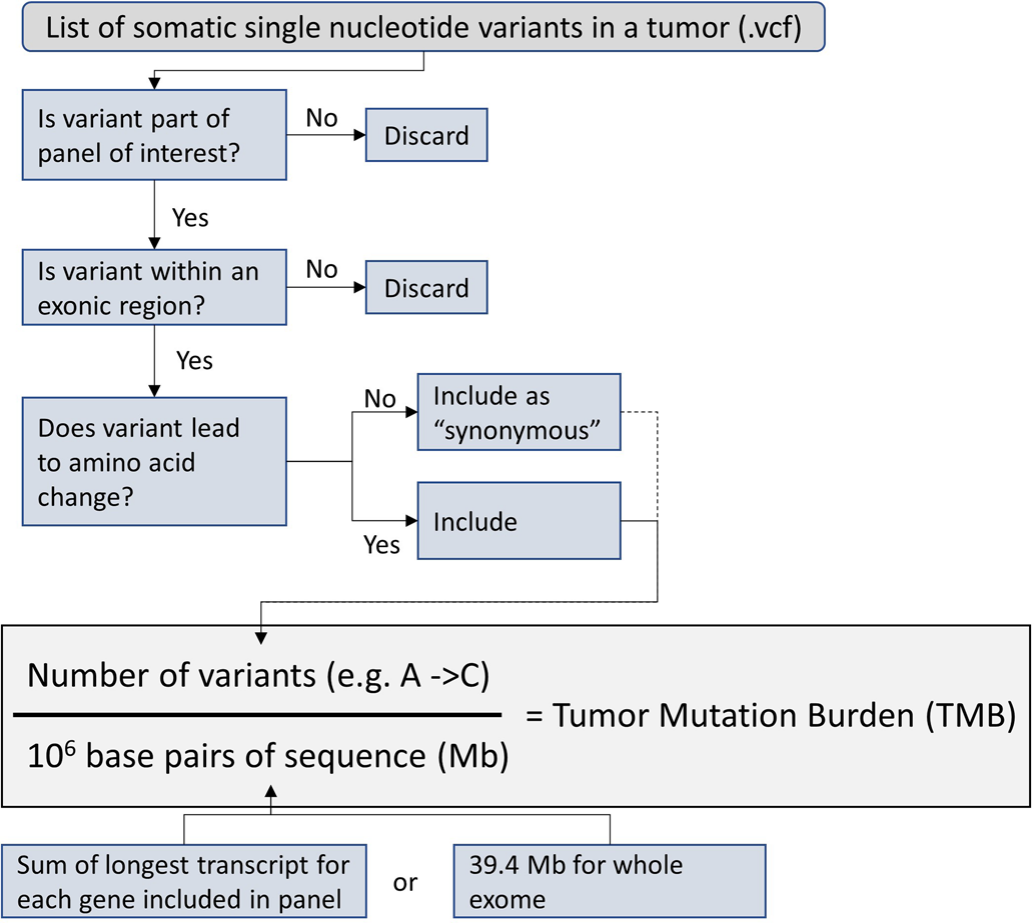
Graphical representation of in silico determination of TMB from variant lists.

Variant counting was performed by using variant annotations within an individual patient tumor’s VCF file. Variants were included if they met the relevant variant inclusion criteria (ie, synonymous vs non-synonymous single nucleotide changes or indels) and occurred within a coding exon of a gene included in the panel of interest or anywhere within the coding portion of the exome in the instance of whole exome sequence (WES) derived TMB (figure 1).

Panel size was estimated by summation of the longest transcript of each gene included in a panel as found in the National Center for Biotechnology Reference Sequence (RefSeq) collection. The exome size was estimated by summation of all coding exons of all genes found within RefSeq. The estimated exome size of 39.4 Mb is within ten percent of previously published determinations of exome size.^10–12^ For this comparative analysis, the lists of genes analyzed in six commonly used clinical NGS assays were used, including: (1) the FoundationOne CDx assay (FM) (324 genes, Foundation Medicine, Cambridge, Massachusetts, USA), (2) the TruSight Tumor 170 (TsT170) assay kit (170 genes, Illumina, San Diego, California, USA), (3) the TruSight Tumor 500 assay (TsT 500) kit (500 genes, Illumina), (4) the Tempus xT assay (596 genes, Tempus, Chicago, Illinois, USA), (5) the MSK Impact assay (MSK) (468 genes, Memorial Sloan Kettering Cancer Center, New York, New York, USA) and (6) the University of California at San Diego (UCSD) Solid Tumor Mutation Panel (STMP) (397 genes, UC San Diego Health, San Diego, California, USA).

Data analysis was performed with R studio (rstudio.com). Regression analysis was performed with the mcReg regression analysis package (cran.r-project.org/web/packages/mcr). Survival analysis and HR calculations were performed with the survminer package (cran.r-project.org/web/packages/survminer). Graphs were created using the ggplot function within the tidyverse package (cran.r-project.org/web/packages/tidyverse). Other figures were created using the Microsoft Office suite of products (Redmond, Washington, USA).

### Results

#### Quantitative impact of inclusion of synonymous variants and gene sampling on TMB

In silico determination of TMB for samples in TCGA was performed as described in the Methods section and summarized in (figure 1). Individual tumor types were shown to have significantly different median TMB values (online supplementary figure 1) consistent with previous analyses of the TCGA data set.^8^

10.1136/jitc-2020-000613.supp1 Supplementary data

The redundancy of the genetic code allows for nucleotide variations that do not lead to amino acid changes in the translated protein. Although these variants may have biological impact, they are largely thought to be clinically silent and, thus, are often referred to as ‘silent’ or ‘synonymous’ variants. Some laboratories include synonymous variants in the calculation of TMB while others do not.

**Figure 2:**
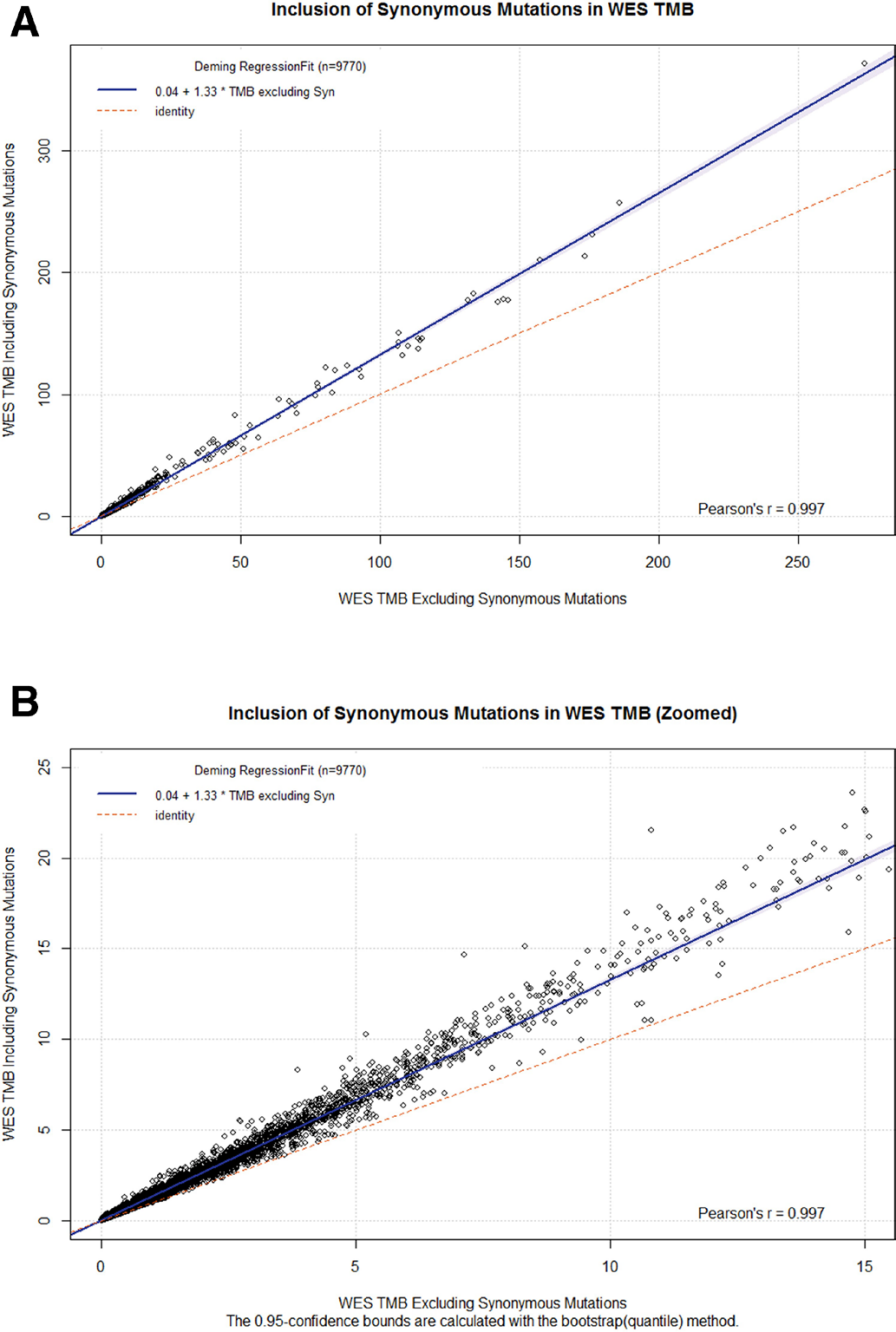
(A) Deming regression between WES-based TMB including synonymous variants (y axis) versus WES-based TMB excluding synonymous variants (X axis). (B) Zoomed version of (A) with X axis truncated at 15 variants/Mbase. TMB, tumor mutation burden; WES, whole-exome sequence.

To measure the impact of synonymous variants on WES TMB, we performed regression analysis on WES TMB values calculated with and without the inclusion of synonymous variants(figure 2A, B). Regression analysis showed a high Pearson’s r (>0.99), indicating a nearly perfect linear correlation between the two methods. Additionally, the slope indicates that, of all nucleotide variants observed, 75% (1/1.33) are non-synonymous while the other 25% will be synonymous. The observed ratio of synonymous to non-synonymous variants is consistent with those previously published.^13^ Bland-Altman plots of WES TMB with and without inclusions of synonymous variants show the magnitude of the difference between the two methods (online supplementary figure 2). Focusing on values near the approximate clinical decision range of TMB <20 (table 1) shows that the difference between the two methods is frequently in the 5–10 variants/Mb range (online supplementary figure 3).

10.1136/jitc-2020-000613.supp2 Supplementary data

10.1136/jitc-2020-000613.supp3 Supplementary data

**Figure 3:**
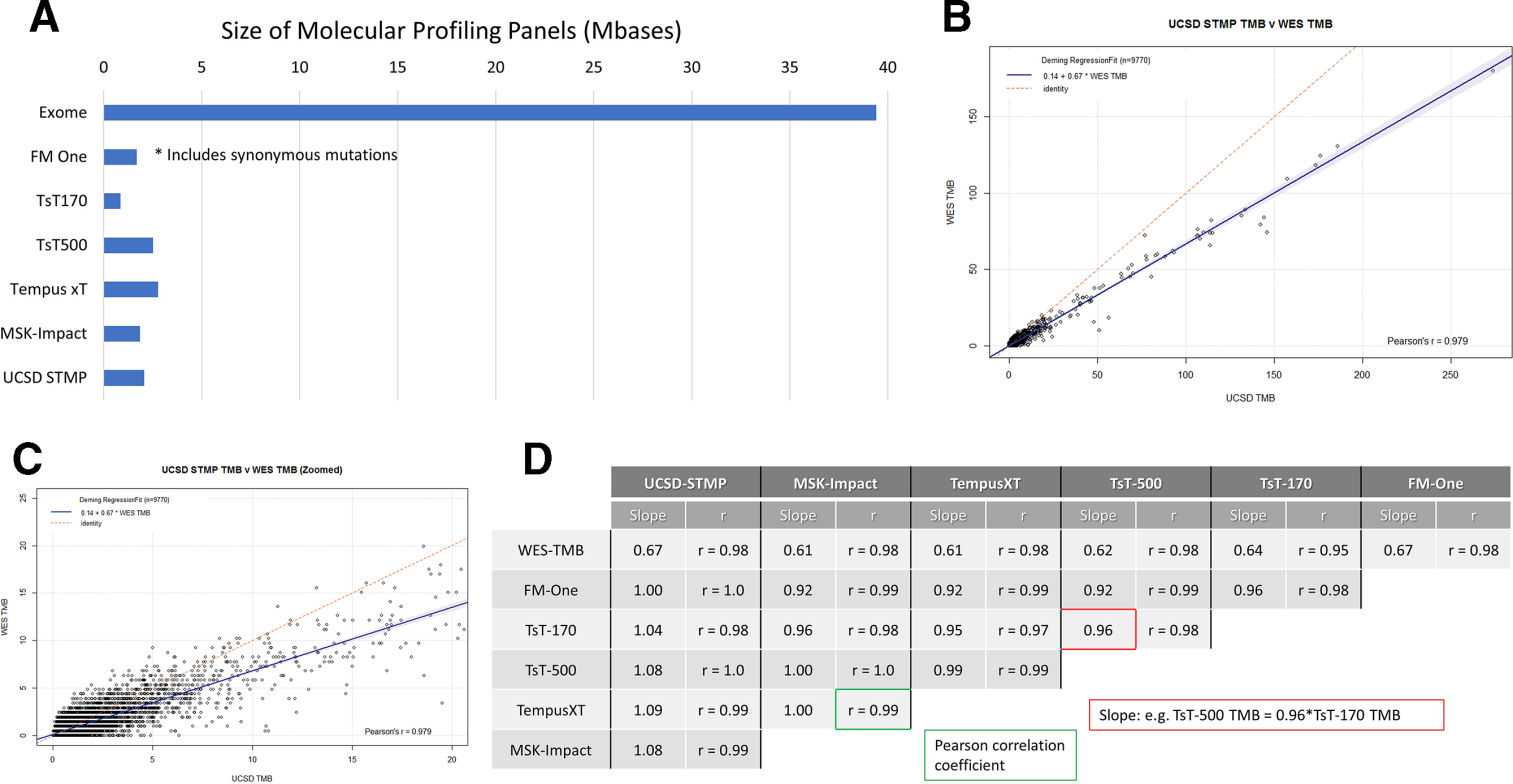
(A) Size of molecular profiling panels in Mbases (10^6^ base pairs). FM One, FoundationOne CDx assay (324 genes, Foundation medicine, Cambridge, MA); TsT170, the TruSight tumor 170 assay kit (170 genes, Illumina, San Diego, California, USA); TsT500, the TruSight tumor 500 assay kit (500 genes, Illumina); Tempus xT. the Tempus XT assay (596 genes, Tempus, Chicago, Illinois, USA); MSK-Impact, the MSK impact assay (468 genes, Memorial Sloan Kettering cancer Center, New York, New York, USA); UCSD STMP, the UCSD Solid Tumor Mutation Panel (397 genes, UC San Diego Health, San Diego). Of note, the FoundationOne panel includes synonymous variants. (B) Deming regression between WES based TMB including synonymous variants (X axis) vs UC San Diego Solid Tumor Mutation Panel (UCSD STMP) panel based TMB (Y axis). (C) Zoomed version of (B) with X axis truncated at 20 variants/Mbase. (D) Pearson correlation coefficients and regression line slopes between WES-TMB and multiple panel based TMB determinations. STMP, Solid Tumor Mutation Panel; TMB, tumor mutation burden; WES, whole-exome sequence.

WES of tumor specimens is rarely performed in routine clinical practice because of cost considerations and lack of well-defined clinical utility. Instead, molecular profiling of tumors is performed by analyzing a subset of the genome defined by a panel of clinically relevant genes. The sizes of six representative panel-based assays in current clinical use, ranging from 0.5 to 3 Mb, represent less than ten percent of the whole exome (figure 3A). These gene panels consist of unique gene sets with varying degrees of overlap between panels (online supplementary figure 4).

10.1136/jitc-2020-000613.supp4 Supplementary data

To measure the impact of gene panel content differences as well as inclusion or exclusion of synonymous mutations, we performed regression analysis on TMB derived from each panel compared with WES TMB calculated without synonymous mutations. Regression analysis comparing the UCSD STMP derived TMB with WES TMB showed a high Pearson’s r (>0.97) indicating a nearly perfect linear relationship (figure 3B, C). The slope of 0.67 indicates that UCSD STMP derived TMB is typically 2/3 that of WES derived TMB. Slope and Pearson’s r values for regression analysis between multiple panels and WES derived TMB are shown in figure 3D. These data indicate that TMB from the panels examined is strongly correlated with WES TMB (Pearson’s r>0.95) with a slope of approximately 0.65 (0.61–0.67). Of key importance, regression analysis between panels showed high correlation (Pearson’s r 0.91–1.0) and slopes of approximately 1 (0.92–1.09) indicating that panel derived TMB measurements are analytically equivalent.

### Correlation of TMB with overall survival

TMB is primarily used as a marker for predicting response to immune checkpoint inhibitor therapy. However, others have demonstrated a positive correlation between TMB and survival in the absence of checkpoint inhibitor therapy for breast adenocarcinoma^14^ and melanoma^15^ and a negative correlation in non-small-cell lung cancer (NSCLC).^16^ Data for patients within the TCGA data set were largely collected prior to FDA approval of checkpoint inhibitor therapy. Examination of clinical data for all tumor types indicates that only two patients with cutaneous melanoma received ipilumimab treatment as part of a clinical trial. We sought to ascertain if TMB is correlated with overall survival (OS) using the publically available clinical data resource of the TCGA data set.^17^

**Figure 4:**
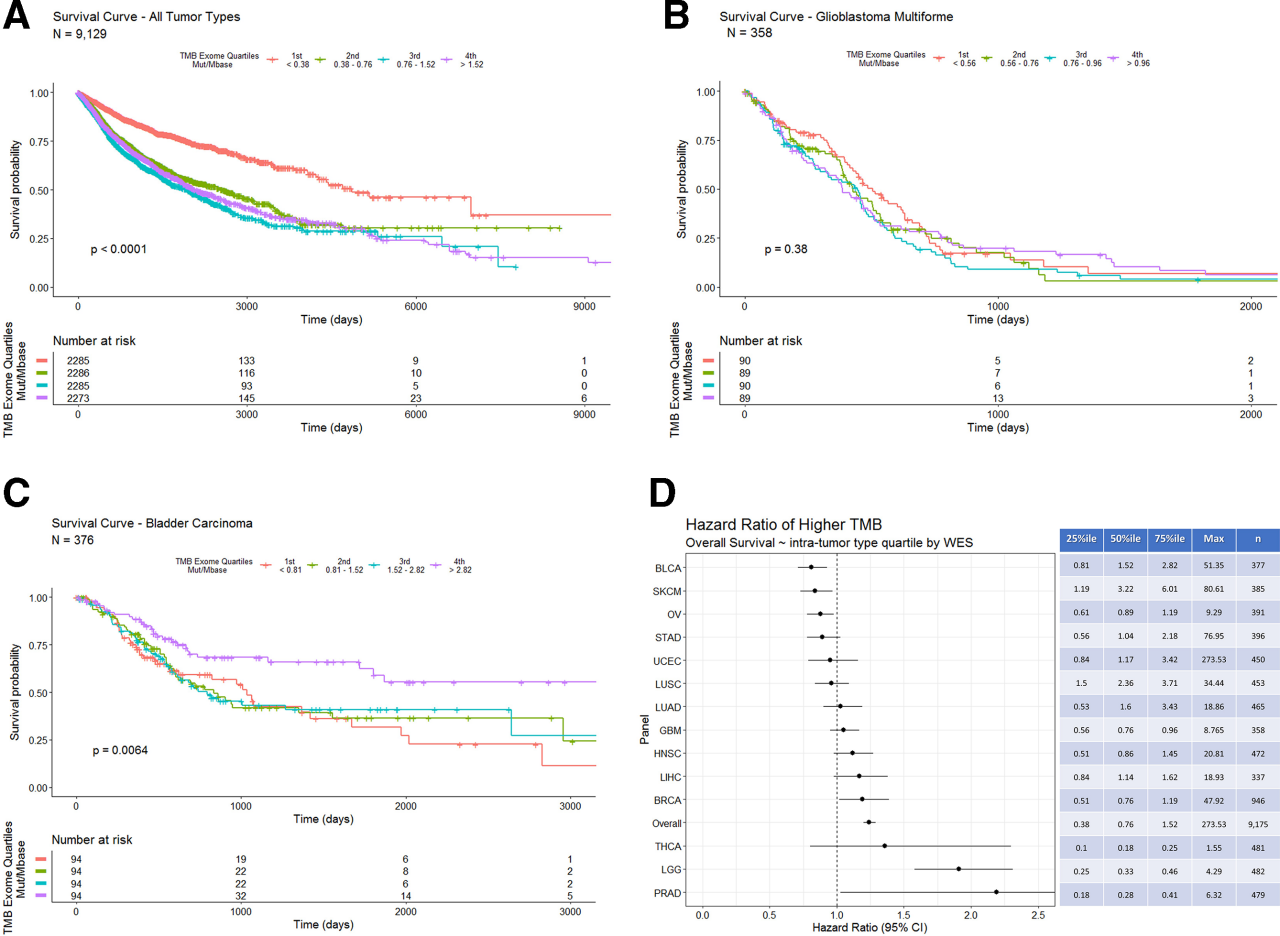
(A) Overall survival in days for all tumor types grouped by WES derived TMB quartiles (the fourth quartile represents the highest 25% of calculated TMB values). Patients with tumors in the lowest TMB quartile (orange line) show a longer overall survival. (B) Overall survival in days for bladder carcinoma grouped by intra-tumor type WES derived TMB quartiles. Patients with tumors in the highest TMB quartile (purple line) show a longer overall survival. (C) Overall survival in days for glioblastoma multiforme grouped by intra-tumor type WES derived TMB quartiles. (D) COX proportional HRs with 95% CIs segmented by tumor type WES TMB intratumor type TMB quartiles. The table on the right shows quartile cut-offs and maxima of WES-TMB for each tumor type as well as the number of samples included. TMB, tumor mutation burden; WES, whole-exome sequence.

Survival analysis of pan-tumor data shows an inverse correlation of TMB derived from WES with OS, that is, patients with higher TMB tend to have a shorter survival time (figure 4A—tumor type composition of TMB quartiles included in online supplementary figure 5). Subgroup analysis indicates that glioblastoma multiforme shows no correlation between TMB and survival (figure 4B). In contrast, bladder carcinoma has a positive correlation between TMB and survival (figure 4C). Of note, only nine patients with bladder carcinoma received BCG therapy, which has an immune mediated mechanism of action analogous to that of checkpoint inhibitor therapy.^18^ Analyses of HRs and TMB quartile indicate differing correlations between TMB and OS (figure 4D). Survival analyses for all tumor types with n>300 are available in online supplementary figures 7–18.

10.1136/jitc-2020-000613.supp5 Supplementary data

10.1136/jitc-2020-000613.supp6 Supplementary data

10.1136/jitc-2020-000613.supp7 Supplementary data

10.1136/jitc-2020-000613.supp8 Supplementary data

10.1136/jitc-2020-000613.supp9 Supplementary data

10.1136/jitc-2020-000613.supp10 Supplementary data

10.1136/jitc-2020-000613.supp11 Supplementary data

10.1136/jitc-2020-000613.supp12 Supplementary data

10.1136/jitc-2020-000613.supp13 Supplementary data

10.1136/jitc-2020-000613.supp14 Supplementary data

10.1136/jitc-2020-000613.supp15 Supplementary data

10.1136/jitc-2020-000613.supp16 Supplementary data

10.1136/jitc-2020-000613.supp17 Supplementary data

**Figure 5:**
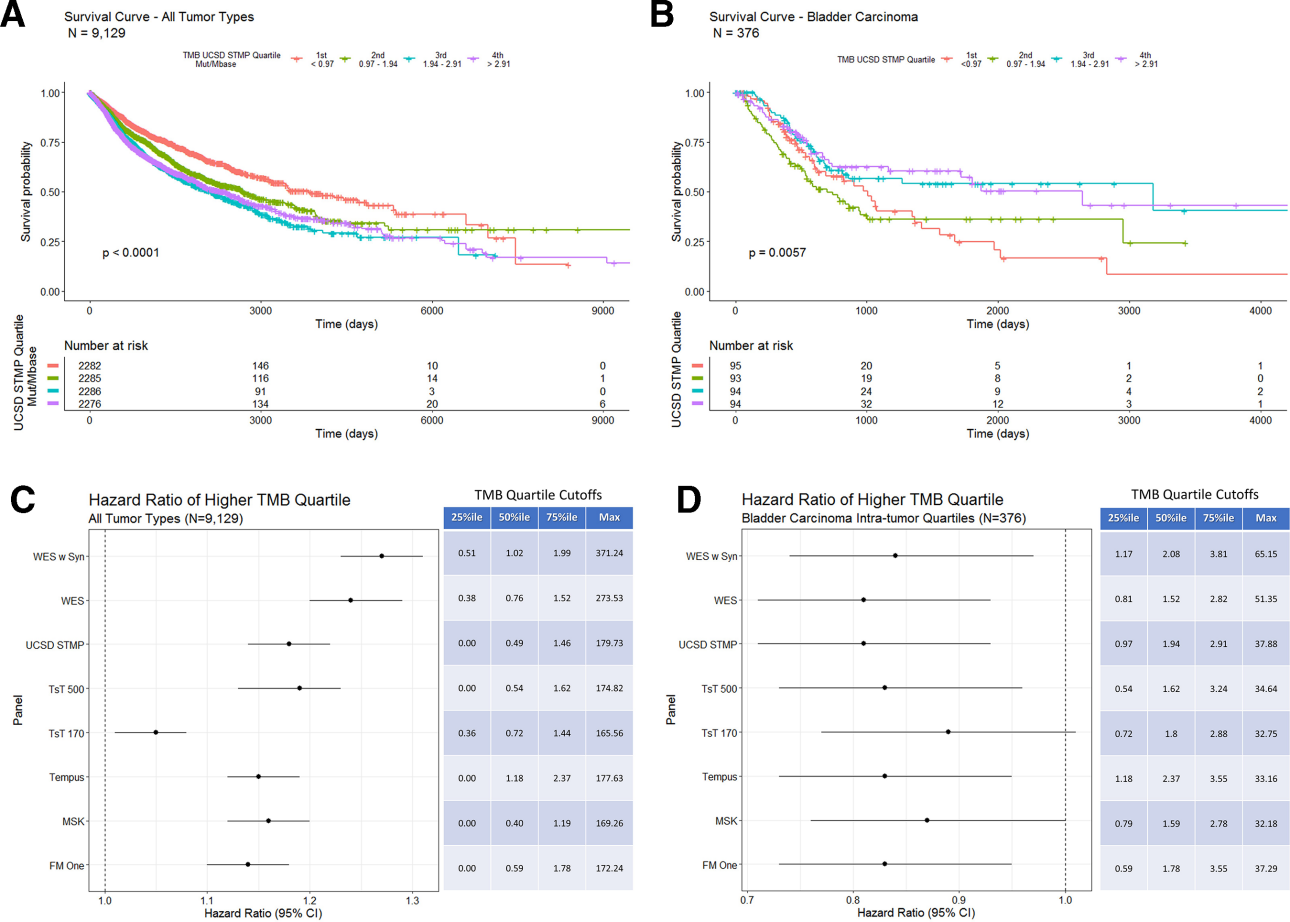
(A) Overall survival in days for all tumor types grouped by UCSD STMP derived TMB quartiles. Patients with tumors in the lowest TMB quartile (orange line) show a longer overall survival. (B) Overall survival in days for bladder carcinoma grouped by intratumor type UCSD STMP derived TMB quartiles. Patients with tumors in the two highest TMB quartiles (purple and blue lines) show a longer overall survival. (C) COX proportional HRs for TMB derived from the indicated panels across all tumor types. The table indicates percentile cut-off points for each TMB calculation method. (D) COX proportional HRs for TMB derived from the indicated panels within bladder carcinoma only. The table indicates intratumor type percentile cut-off points for each TMB calculation method. STMP, solid tumor mutation panel; TMB, tumor mutation burden; WES, whole-exome sequence.

Using the gene list from the UCSD STMP as an example to calculate panel derived TMB also shows an inverse correlation with OS (figure 5A—tumor type composition of TMB quartiles included in online supplementary figure 6) similar to that seen with WES TMB (figure 4A). Additionally, bladder carcinoma shows a positive correlation between UCSD STMP based TMB and survival (figure 5B). HR analysis of TMB determined by multiple computational methods for all tumor types shows a consistent inverse correlation with OS regardless of method (figure 5C). Similarly, HRs of TMB by multiple computation methods for bladder cancer alone shows a consistent correlation with OS regardless of method (figure 5D). The persistence of the correlation between TMB and survival is largely preserved despite differing methods of calculating TMB, suggesting that the differing methods are both analytically and clinically equivalent.

10.1136/jitc-2020-000613.supp18 Supplementary data

## Discussion

Molecular characterization of tumors has quickly become standard of care in oncology. Recent survey data indicates that 75% of oncologists across practice types and sizes routinely use NGS data to guide patient care decisions.^19^ Many commercially available and laboratory-developed NGS based tumor profiling assays also include an evaluation of TMB.

TMB measurement requires multiple steps starting with DNA extraction from tissue, followed by sequencing and alignment, variant identification and informatics-based TMB calculations. Given the complexity and cost of TMB determination, harmonization between laboratories is challenging. A recent review of proficiency testing results from the College of American Pathologists for 111 laboratories indicates that NGS-based testing reliably identifies variants across multiple laboratories in more than 98% of samples tested.^20^ Thus, identification of individual variants is unlikely to cause significant differences in TMB determinations between laboratories. Instead, discordance between TMB measurement and interpretation is likely to be secondary to differences in variant inclusion (ie, inclusion vs exclusion of synonymous variants), gene panel size and TMB cut-offs used for clinical decision making. The experiment described here addresses the impact of panel size and variant inclusion criteria by calculating TMB from a controlled set of variant lists using differing informatics methods. It should be noted that sample preparation and sequencing methods designed for high sensitivity and specificity in most clinical labs today are likely different from the research methods of the TCGA.

TMB is an aggregate measure of variants identified from sequencing nucleic acids obtained from tumor bulk consisting of both tumor cells and non-neoplastic tumor-associated cells. The current hypothesis that TMB is a surrogate marker for tumor neoantigen formation is widely accepted and consistent with the correlation between high TMB and response to checkpoint inhibitor therapy.^21^ Given the expectation that TMB is correlated with amino acid changes at the protein level of expressed genes, synonymous mutations should not be included in TMB assessments. Our data indicate that the inclusion of synonymous variants in the calculation of TMB from WES creates a linear bias (figure 2A, B). However, including synonymous variants in TMB derived from gene panels, such as the FoundationOne CDx assay, does not create a discordance between similarly sized panels that exclude synonymous variants (figure 3).

WES is not currently used for routine tumor genome profiling due to cost and clinical utility considerations. Thus, selective gene panels are commonly used for routine patient care. To date, only limited efforts to perform simultaneous WES and smaller panel TMB assessments have been performed (n=29) and results were not definitive (linear regression R^2^=0.75).^22^ Previously published methods of estimating the impact of panel size and content on TMB have used stochastic filtering methods where random exome sampling of similar size to the gene panel was performed to assess the correlation between WES TMB and panel TMB.^23–25^ In contrast, the methods used here for panel TMB estimation used only those genes contained in commonly used panels.

The correlation between panel based and WES TMB values is linear. Thus, in our data set, we see a preservation of the clinical correlation between TMB derived from WES and OS when method-specific quartile values are used to segment patients. TMB values based on any of the panels investigated in this study provide an equivalent estimate of the mutation burden of a tumor; however, decisions based on an individual patient’s TMB value must take into account the source of the data used to calculate the patient’s TMB (eg, UCSD STMP) and the population data source used to inform the decision (WES TMB on a clinical trial population). Reassuringly, the correlation between panel-based TMB values showed slopes of 0.9–1.1 (figure 3D), indicating that the absolute TMB value is comparable between panels.

As with most laboratory values, TMB is often reported with an associated reference range to guide clinical interpretation. The Clinical Genomics Laboratory at UC San Diego reports the calculated TMB value along with a designation of ‘high’, ‘medium’ and ‘low’ based on published TMB population distributions from glioblastoma multiforme, the most frequently profiled tumor type in the laboratory.^26^ While the value of TMB is analytically equivalent between the methods evaluated, it is important to note that interpretation cut-offs are dependent on the clinical utilization of TMB (eg, predicting response to immunotherapy vs patient prognosis) and the specific tumor type. The patient cohort in this analysis did not receive immunotherapy, thus, cut-offs to predict response to immunotherapy cannot be derived from our analysis. As additional data iare made available, tumor-specific reporting ranges informing response to checkpoint inhibitor therapy, patient prognosis, or both would be preferable. Additional data collection and analysis is needed to establish these ranges.

Our analysis of the correlation between TMB and OS in the absence of checkpoint inhibitor therapy suggests that TMB may be a confounding factor in response to therapy and survival analysis of checkpoint inhibitor therapy in some cancer types. Other investigations have also shown a correlation between TMB and OS in the absence of checkpoint inhibitor therapy.^14 15^ Consistent with these findings, a recent analysis of data from a prospective trial comparing checkpoint inhibitor therapy to chemotherapy in TMB high NSCLC showed no OS benefit of checkpoint inhibitor therapy despite higher response rates and longer progression-free survival.^27^ Additional prospective clinical trials using TMB as a biomarker will continue to clarify its clinical utility.

TMB is hypothesized to be a coarse approximation of neoantigen formation. Its relevance to the underlying molecular processes driving and perpetuating tumorigenesis is not clearly known. Our finding that TMB is consistently higher in WES based TMB compared with panel-based TMB is intriguing in that it suggests that the panels analyzed are sampling portions of the genome with lower than average mutation burden. These findings have also been observed in an independent analysis of the TCGA data set.^28^ Currently, gene panels are typically designed to identify actionable point mutations, indels or copy number variants and not to assess overall TMB. Further investigation may show that mutations in some genes are more readily tolerated than others because of functional redundancy, decreased neoantigen presentation due to loss of major histocompatibility complex (MHC) expression or combinations of other mechanisms. Novel biomarkers, such as predictions of binding and presentation of neoantigens on specific MHC alleles, may provide superior predictive value for prognosis or response to therapy.^29 30^ As our understanding of immunoncology continues to increase the utility of TMB and other biomarkers will be clarified.
