# Supplementary material for: Comparison of commonly used solid tumor targeted gene sequencing panels for estimating tumor mutation burden shows analytical and prognostic concordance within the cancer genome atlas cohort
Source: J Immunother Cancer. 2020 Mar 26;8(1):e000613. doi: 10.1136/jitc-2020-000613 (PMC7174068; doi:10.1136/jitc-2020-000613)
Supplement: Supplementary data [file jitc-2020-000613supp002.pdf]

S2

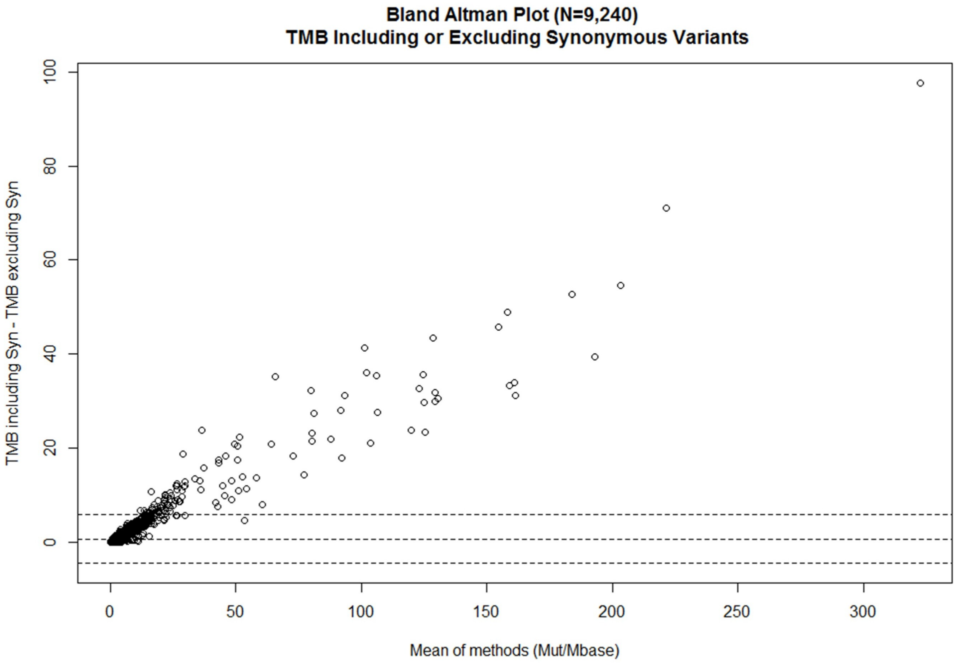

Difference plot showing mean of TMB including and excluding synonymous variants (x axis) and the difference between each method (y axis)
