# Supplementary figures and images for: Comparison of commonly used solid tumor targeted gene sequencing panels for estimating tumor mutation burden shows analytical and prognostic concordance within the cancer genome atlas cohort
Source: J Immunother Cancer. 2020 Mar 26;8(1):e000613. doi: 10.1136/jitc-2020-000613 (PMC7174068; doi:10.1136/jitc-2020-000613)

S3

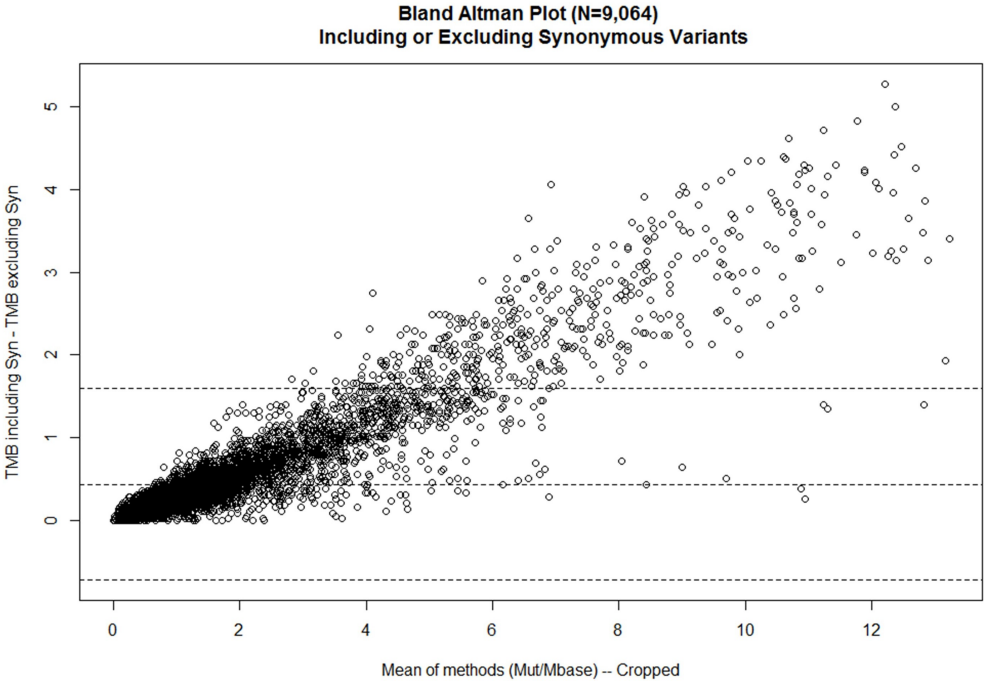

Zoomed difference  
plot with x axis  
truncated to values  
less than 15  
variants/Mbase

Supplement: Supplementary data [file jitc-2020-000613supp003.pdf]

S4

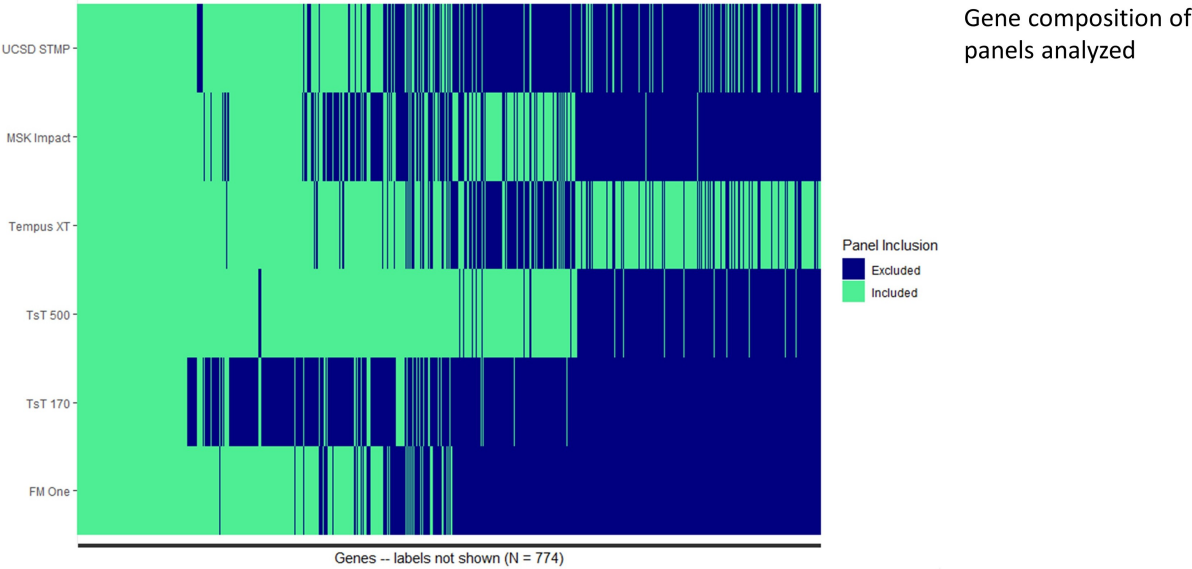

Supplement: Supplementary data [file jitc-2020-000613supp004.pdf]

S7

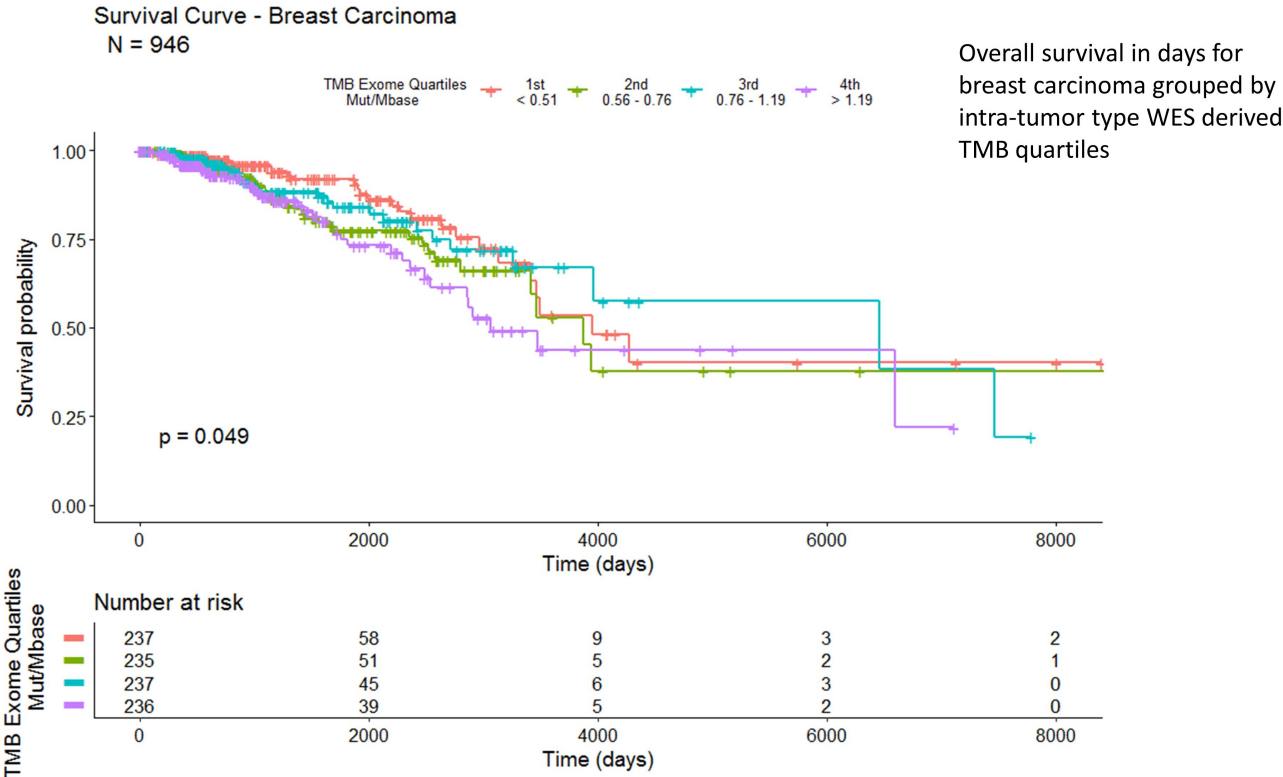

Supplement: Supplementary data [file jitc-2020-000613supp006.pdf]

S8

Survival Curve - Head and Neck Squamous Carcinoma  
N = 472

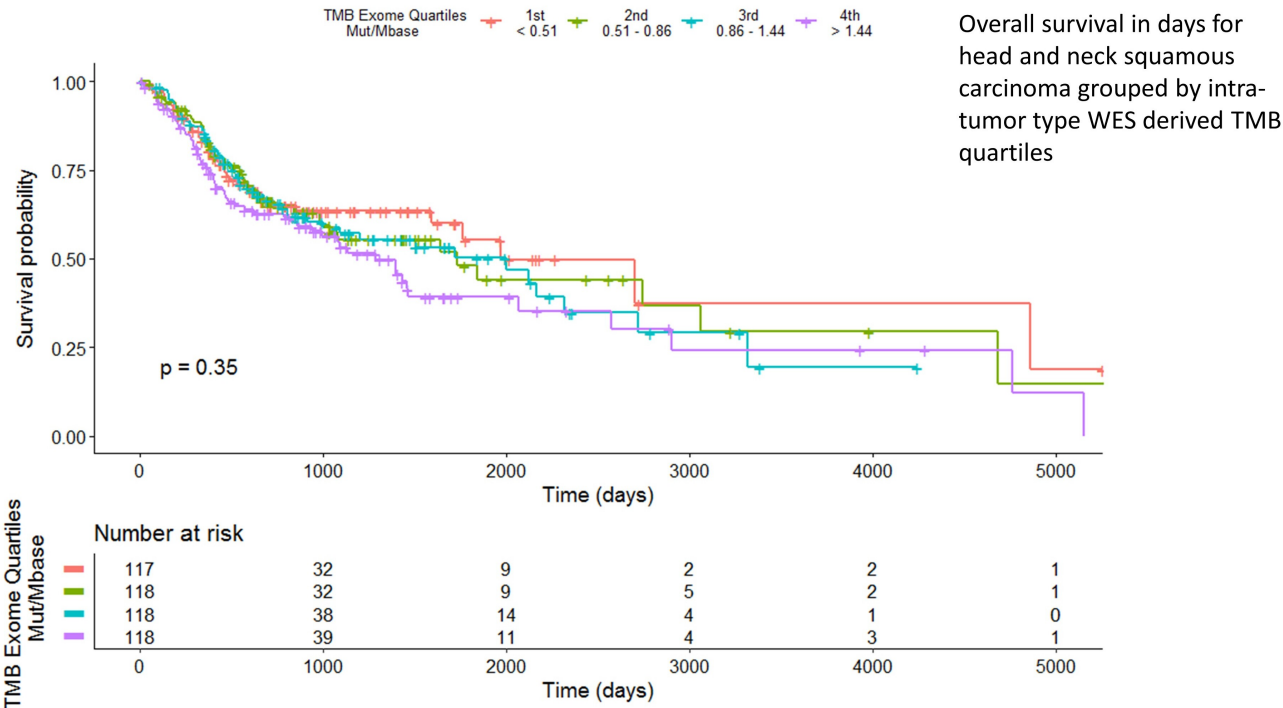

Supplement: Supplementary data [file jitc-2020-000613supp007.pdf]

S9

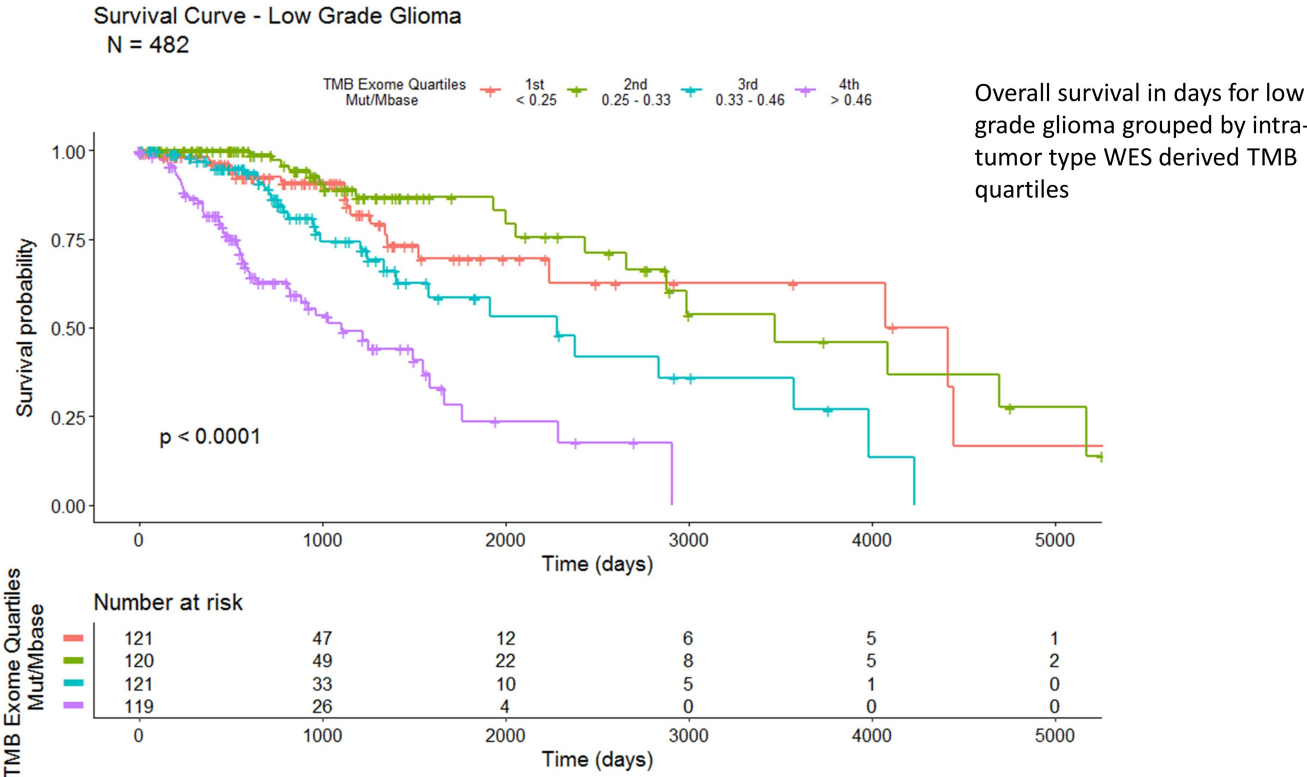

Supplement: Supplementary data [file jitc-2020-000613supp008.pdf]

S10

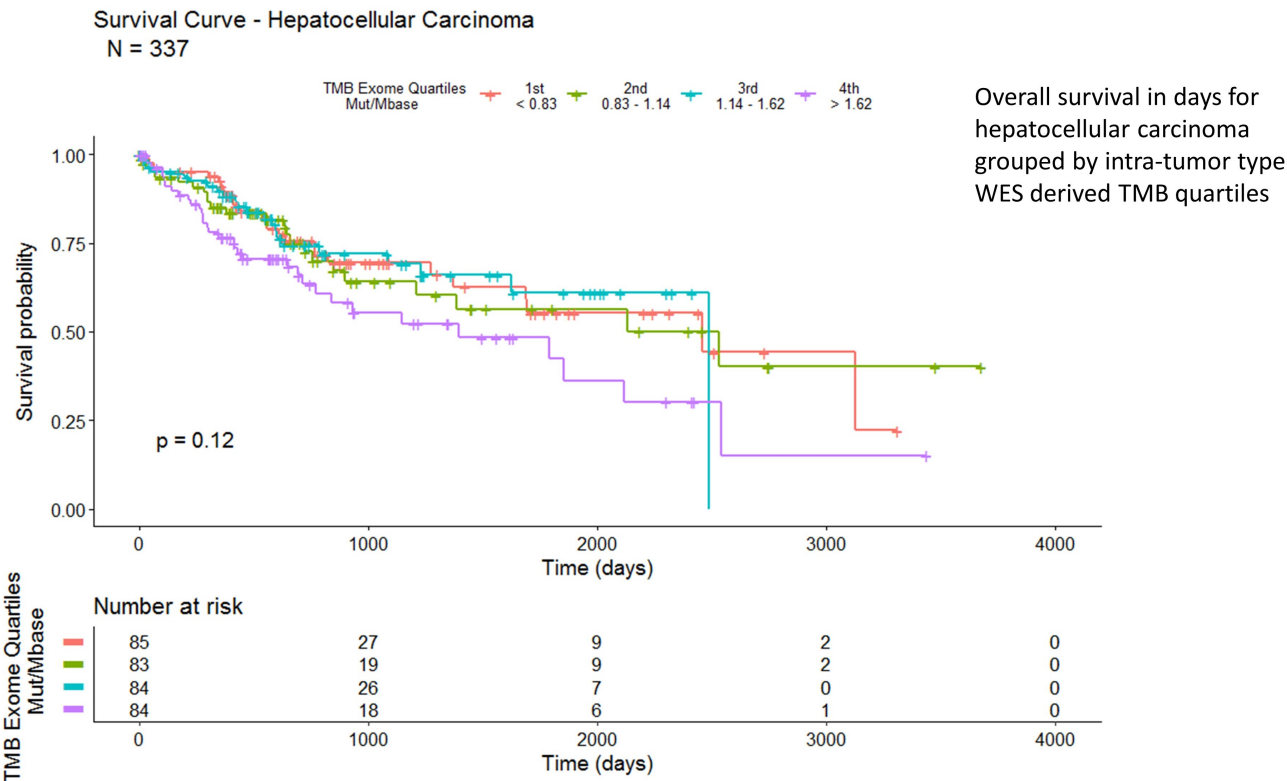

Supplement: Supplementary data [file jitc-2020-000613supp009.pdf]

S11

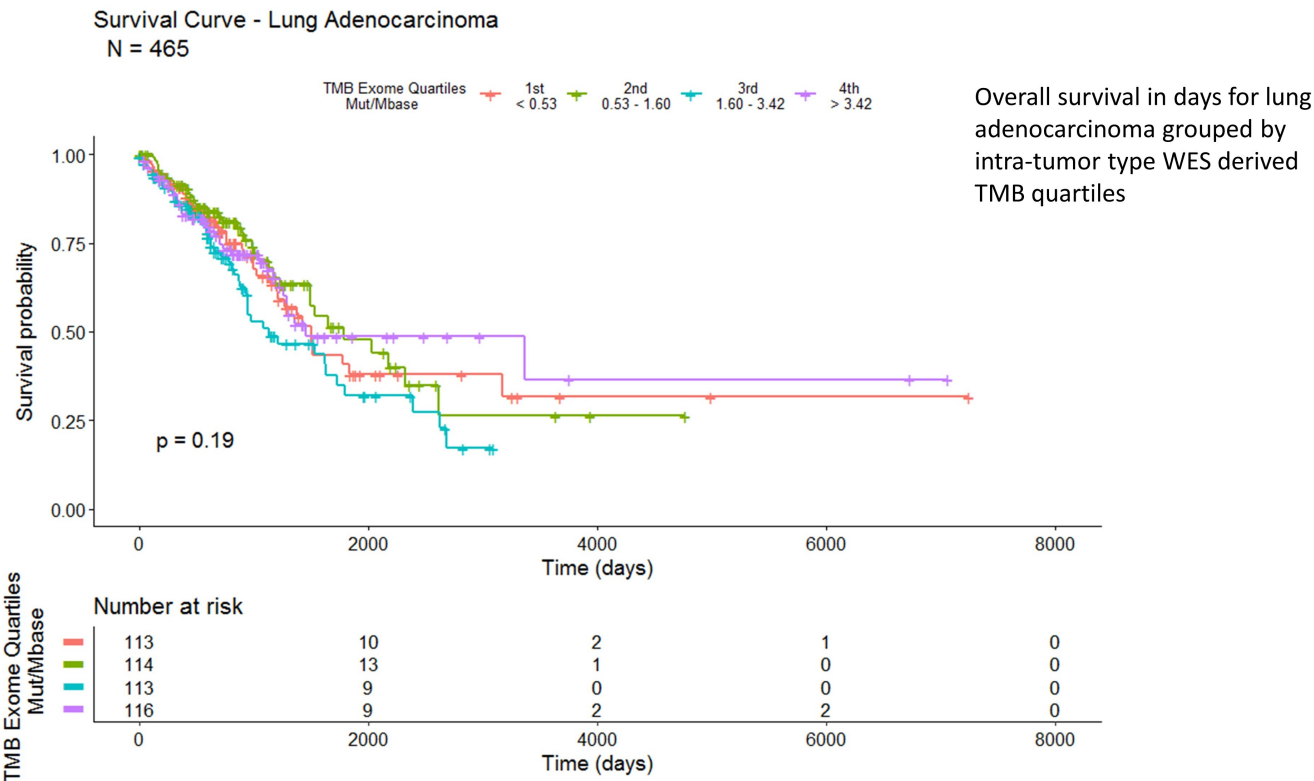

Supplement: Supplementary data [file jitc-2020-000613supp010.pdf]

S12

Survival Curve - Lung Squamous Cell Carcinoma  
N = 453

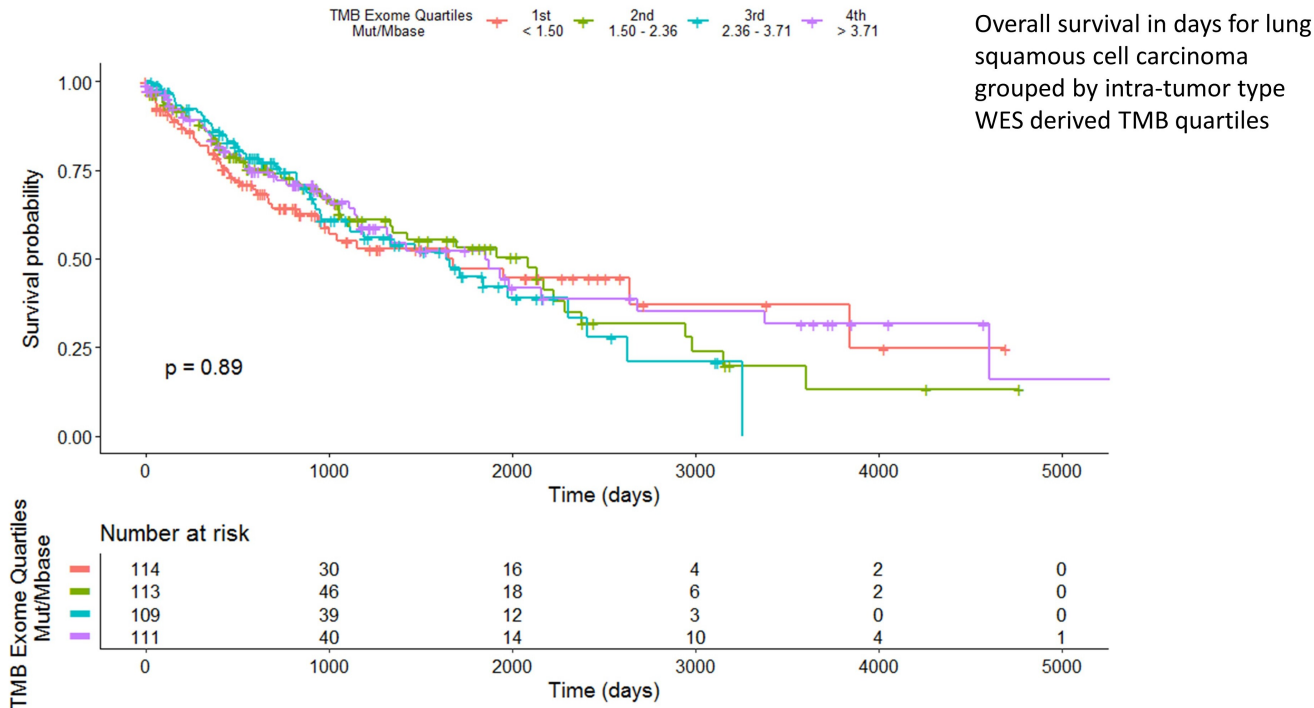

Supplement: Supplementary data [file jitc-2020-000613supp011.pdf]

S13

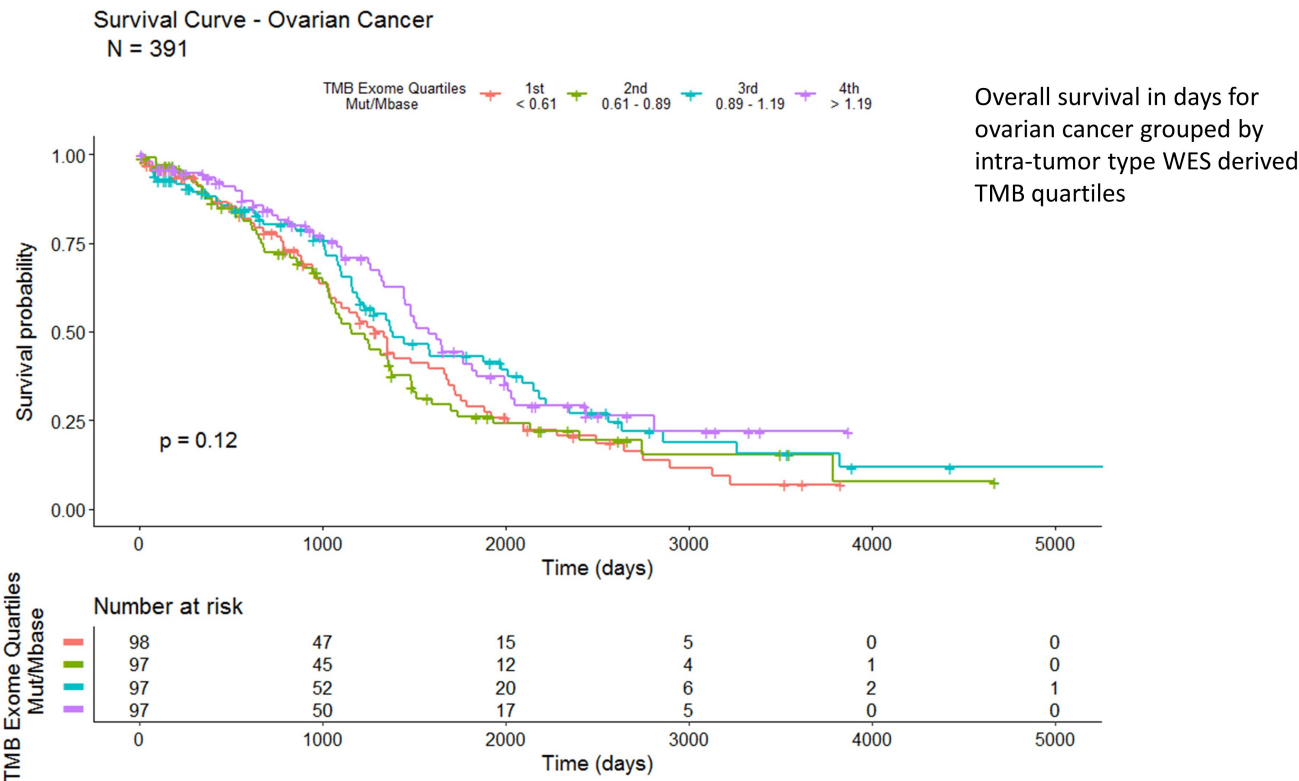

Supplement: Supplementary data [file jitc-2020-000613supp012.pdf]

S14

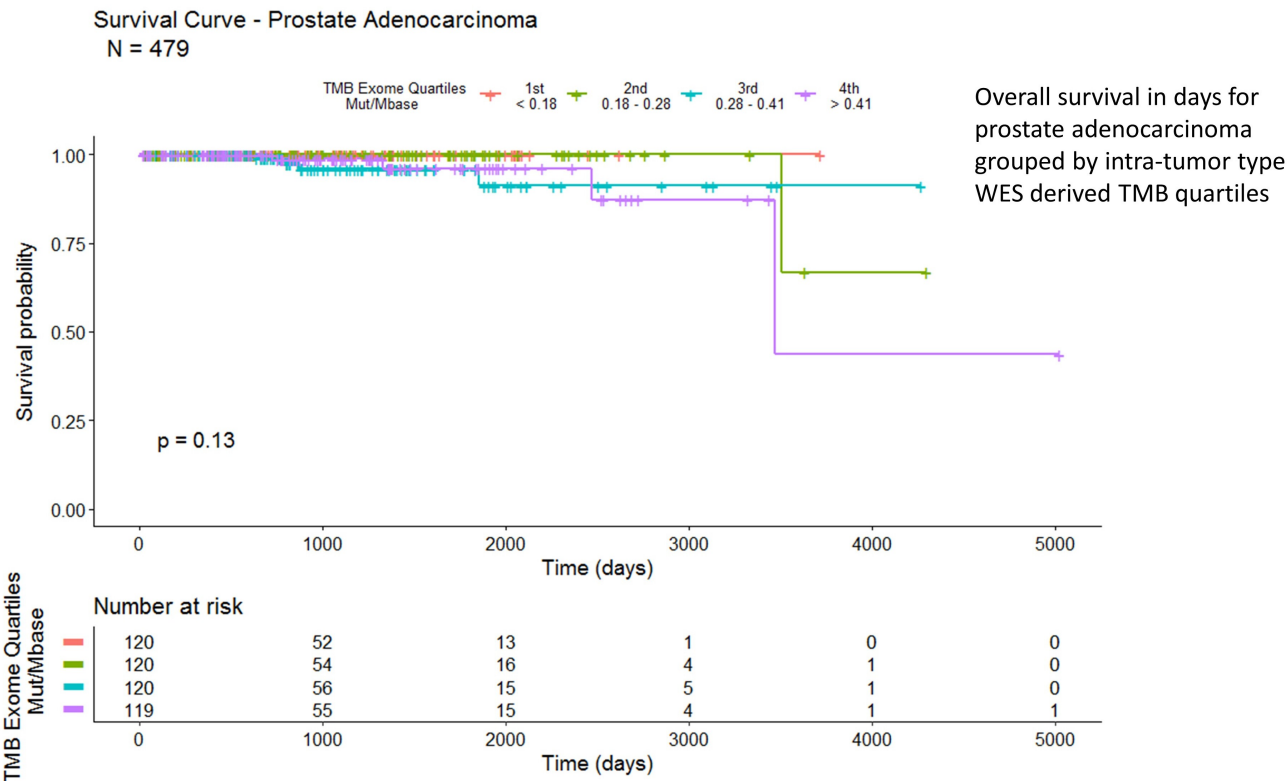

Supplement: Supplementary data [file jitc-2020-000613supp013.pdf]

S15

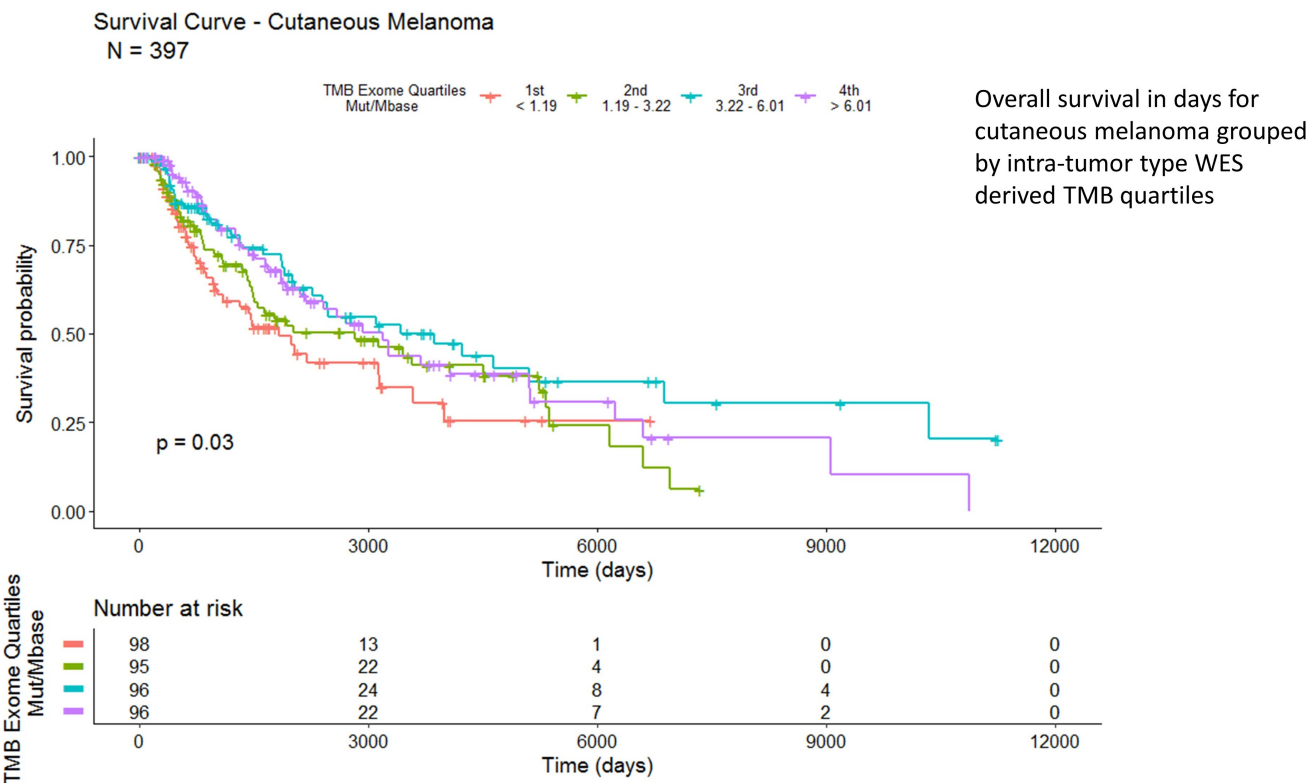

Supplement: Supplementary data [file jitc-2020-000613supp014.pdf]

S16

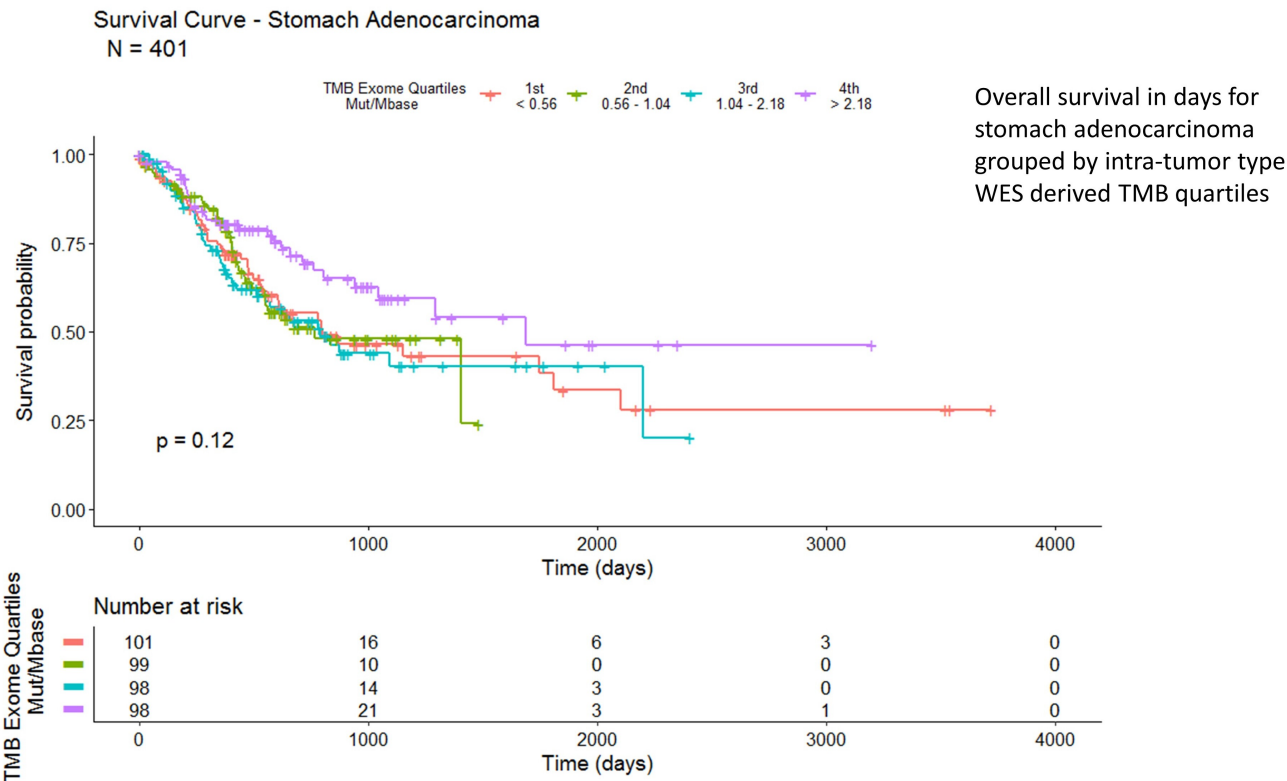

Supplement: Supplementary data [file jitc-2020-000613supp015.pdf]

S17

Survival Curve - Thyroid Carcinoma  
N = 481

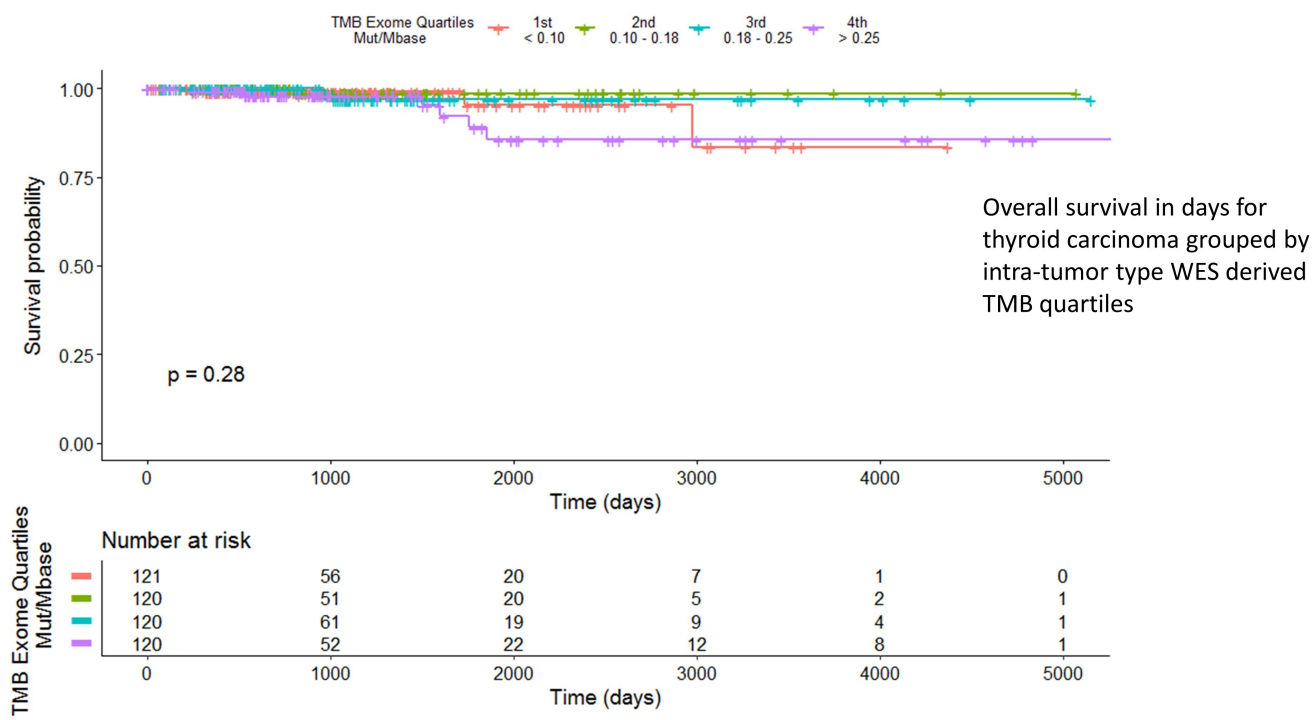

Supplement: Supplementary data [file jitc-2020-000613supp016.pdf]

S18

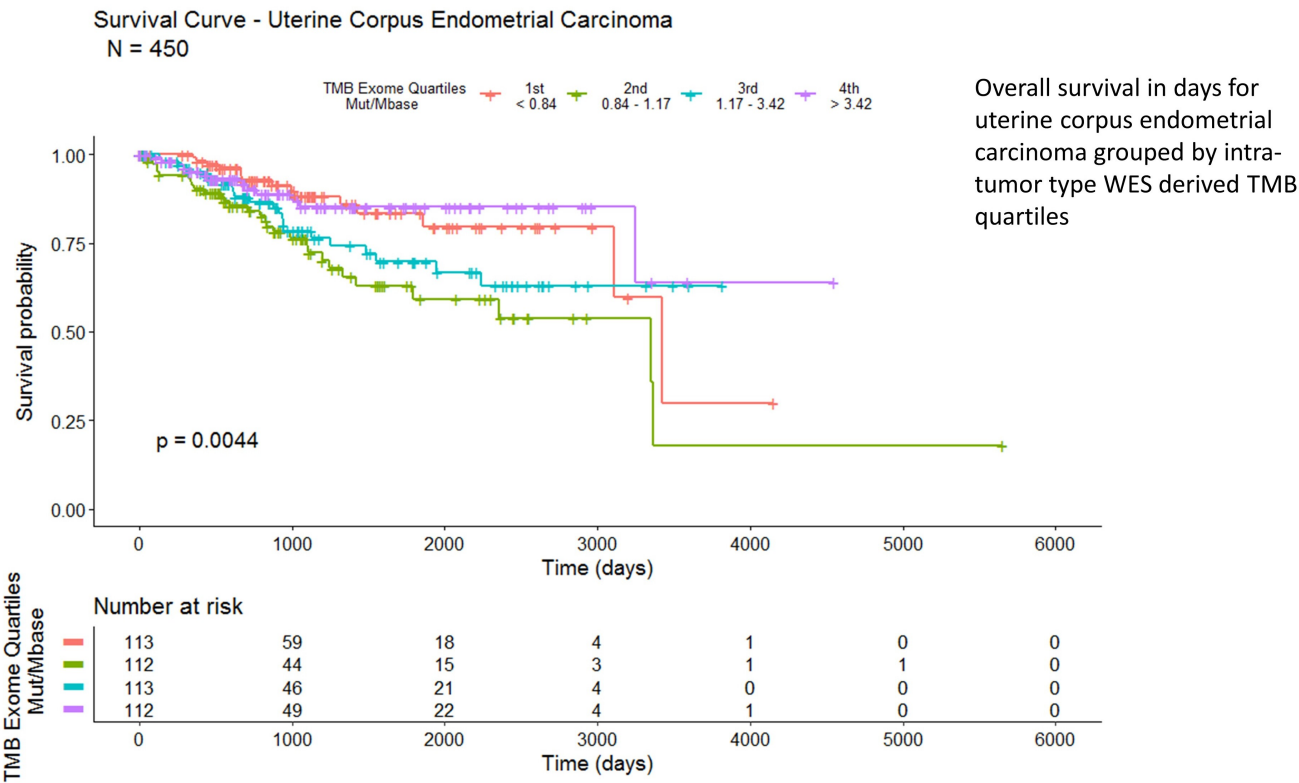

Supplement: Supplementary data [file jitc-2020-000613supp017.pdf]

S6

Tumor Type Composition of Tumor Mutation Burden (TMB) Quartiles  
TMB by UCSD Method

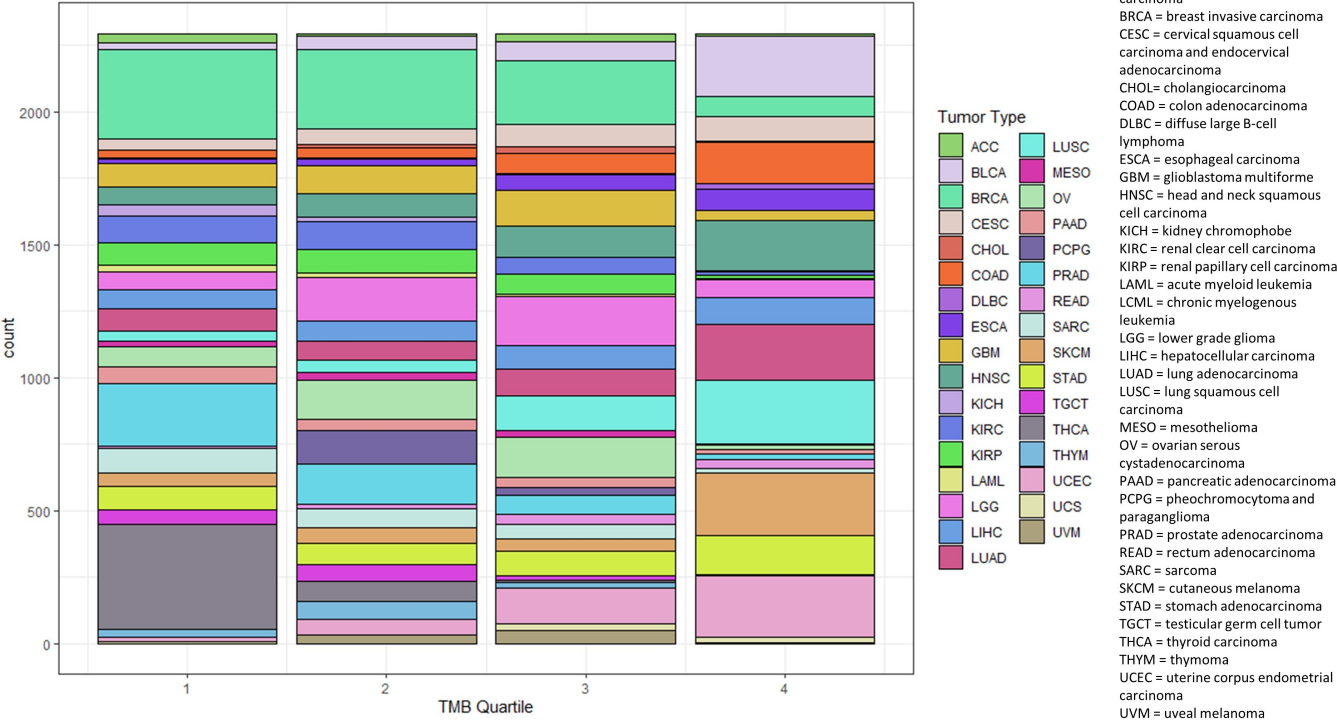

Supplement: Supplementary data [file jitc-2020-000613supp018.pdf]
