# Supplementary material for: Comparison of commonly used solid tumor targeted gene sequencing panels for estimating tumor mutation burden shows analytical and prognostic concordance within the cancer genome atlas cohort
Source: J Immunother Cancer. 2020 Mar 26;8(1):e000613. doi: 10.1136/jitc-2020-000613 (PMC7174068; doi:10.1136/jitc-2020-000613)
Supplement: Supplementary data [file jitc-2020-000613supp005.pdf]

S5

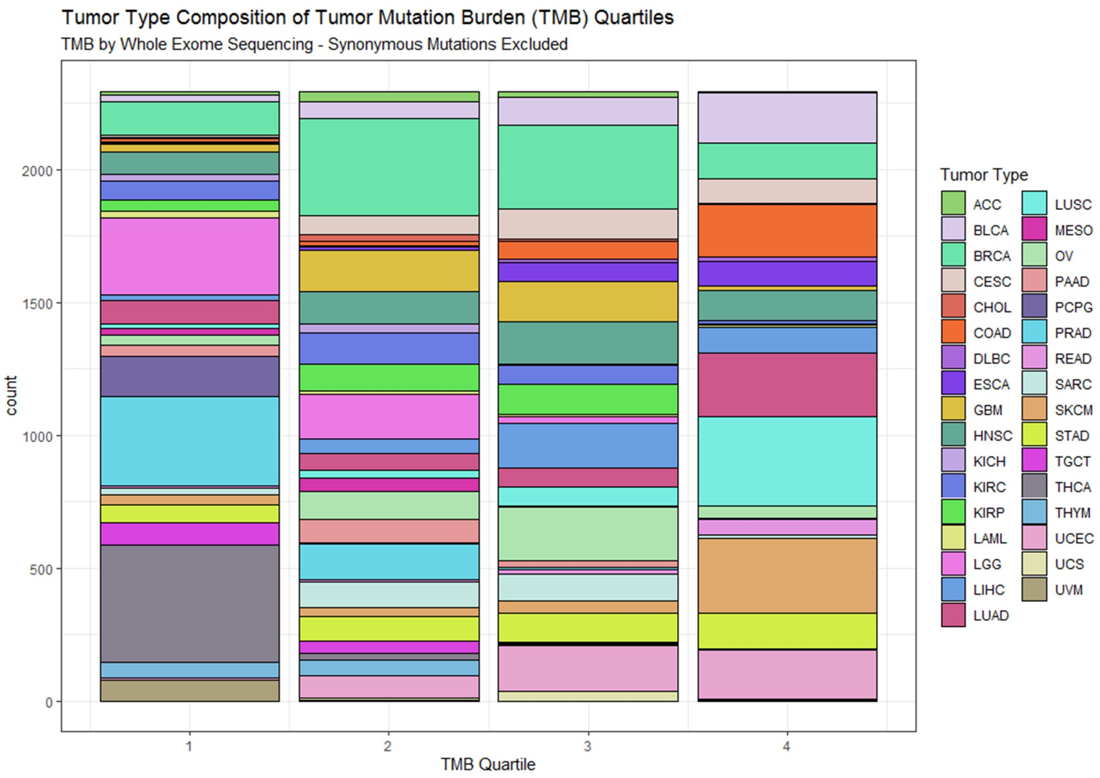

Tumor types:
ACC = adrenocortical carcinoma
BLCA = bladder urothelial carcinoma
BRCA = breast invasive carcinoma
CESC = cervical squamous cell carcinoma and endocervical adenocarcinoma
CHOL= cholangiocarcinoma
COAD = colon adenocarcinoma
DLBC = diffuse large B-cell lymphoma
ESCA = esophageal carcinoma
GBM = glioblastoma multiforme
HNSC = head and neck squamous cell carcinoma
KICH = kidney chromophobe
KIRC = renal clear cell carcinoma
KIRP = renal papillary cell carcinoma
LAML = acute myeloid leukemia
LCML = chronic myelogenous leukemia
LGG = lower grade glioma
LIHC = hepatocellular carcinoma
LUAD = lung adenocarcinoma
LUSC = lung squamous cell carcinoma
MESO = mesothelioma
OV = ovarian serous cystadenocarcinoma
PAAD = pancreatic adenocarcinoma
PCPG = pheochromocytoma and paraganglioma
PRAD = prostate adenocarcinoma
READ = rectum adenocarcinoma
SARC = sarcoma
SKCM = cutaneous melanoma
STAD = stomach adenocarcinoma
TGCT = testicular germ cell tumor
THCA = thyroid carcinoma
THYM = thymoma
UCEC = uterine corpus endometrial carcinoma
UVM = uveal melanoma
